# Post-earthquake Zika virus surge: Disaster and public health threat amid climatic conduciveness

**DOI:** 10.1038/s41598-017-15706-w

**Published:** 2017-11-13

**Authors:** Miguel Reina Ortiz, Nicole K. Le, Vinita Sharma, Ismael Hoare, Edy Quizhpe, Enrique Teran, Eknath Naik, Hamisu M. Salihu, Ricardo Izurieta

**Affiliations:** 1 Department of Global Health, College of Public Health, University of South Florida, Tampa, FL USA; 2 Fundación Raíces, Esmeraldas, Ecuador; 3 Morsani College of Medicine, University of South Florida, Tampa, FL USA; 4 Department of Community and Family Health, College of Public Health, University of South Florida, Tampa, FL USA; 5 Ministerio de Salud Pública del Ecuador, Quito, Ecuador; 6 Colegio de Ciencias de la Salud, Universidad San Francisco de Quito, Quito, Ecuador; 7 Emergency Department, James A Haley VA Hospital, Tampa, FL USA; 8 Department of Family and Community Medicine, Baylor College of Medicine, Houston, TX USA

## Abstract

A recent major earthquake (M7.8), coupled with appropriate climatic conditions, led to significant destruction in Ecuador. Temperature variations, which may be induced by anthropogenic climate change, are often associated with changes in rainfall, humidity and pressure. Temperature and humidity are associated with ecological modifications that may favour mosquito breeding. We hypothesized that the disruptive ecological changes triggered by the earthquake, in the context of appropriate climatic conditions, led to an upsurge in Zika virus (ZIKV) infections. Here we show that, after controlling for climatic and socioeconomic conditions, earthquake severity was associated with incident ZIKV cases. Pre-earthquake mean maximum monthly temperature and post-earthquake mean monthly pressure were negatively associated with ZIKV incidence rates. These results demonstrate the dynamics of post-disaster vector-borne disease transmission, in the context of conducive/favourable climatic conditions, which are relevant in a climate change-affected world where disasters may occur in largely populated areas.

## Introduction

Anthropogenic climate change has led to changing global temperatures, which may be associated with changes in rainfall as well as other important climatic variables like humidity and pressure^1^. It has previously been shown that environmental characteristics, especially temperature and humidity are associated with mosquito life cycle length^2,3^, malaria extrinsic incubation period^3^, dengue incidence^4,5^, emerging infectious diseases^6^ and other vector-borne disease indicators. Briefly, within an appropriate range, increasing temperatures are associated with shorter mosquito life cycles^2^ that can lead to increased vector-borne disease transmission. However, excessively high temperatures might actually negatively impact vector-borne disease epidemiology^7^. In addition, mosquitoes tend to breed in natural or artificial water containers/bodies^2^, which may abound in environmentally-disrupted post-disaster scenarios^8^. Therefore, the unfortunate occurrence of disasters in the context of appropriate temperature and/or humidity/rainfall/pressure may represent the “perfect storm” that will lead to an increase in the public health burden of vector-borne diseases. In this study, we evaluated the effect that a recent powerful earthquake, in the context of the local climatic conditions, had on the reported number of ZIKV cases. The tropics of Ecuador and its recent earthquake experience represent a natural experiment to study this phenomenon.

In April 2016, Ecuador experienced a massive 7.8 M earthquake along its unstable Nazca and South American plates—the strongest seism in almost four decades^9^. It resulted in about 700 deaths and 30,000 injuries, internally displacing an estimated 73,000 people^10,11^. Though it was felt nationwide, the earthquake affected two coastal provinces, Esmeraldas and Manabí, the hardest^11^.

Natural disasters, like earthquakes, are often associated with or followed by serious public health consequences such as increased risk for communicable diseases, including waterborne and vector-borne diseases^12–15^. The 2010 earthquake in Haiti resulted in both immense structural damage and a large cholera outbreak^15^. The massive damages in the aftermath resulted in a devastated infrastructure^15^. People were displaced and crowded into unsanitary camps after losing their homes, where they lacked clean water and sanitation^16^. This allowed for cholera to spread rapidly via contaminated water supplies^15^. Furthermore, after the earthquake, malaria became an even greater public health concern in Haiti^17,18^. Unintentional water collection in building remnants and debris may provide a conducive environment for mosquito breeding^19^. Similar breeding grounds have been reported in partially built structures in Malaysia^20^, which may, in this context, fulfil the same vector-breeding function that debris play in post-disaster scenarios. In addition to unintentional water collections, abandoned and unmaintained pools, especially in lower socio-economic communities, can provide breeding grounds for mosquitoes^21–23^.

After the earthquake in Ecuador, several health conditions, including acute respiratory infections and diarrhoea, were reported^11^. Just as in Haiti, post-earthquake scenarios might have led to altered ecological environments conducive to mosquito proliferation, including *Aedes spp*. (the main vector for Zika virus), causing increased mosquito populations^19,24,25^. Such environments could be associated with rapid and unplanned resettlement, worsening of hygienic and environmental conditions due to significant destruction of water and sanitation infrastructure^19,24,25^. In addition, displaced populations may be at increased risk for vector-borne diseases due to behavioural exposures (i.e. people are forced to spend more time outdoors) as well as housing conditions (i.e. living in temporary shelters without window screens)^24,26^. Other post-earthquake/post-disaster conditions that could lead to increased vector-borne disease transmission include landslides, deforestation, river damming, and rerouting of natural water flows^26^. These populations are also at risk for an increase in psychological disorders and substance abuse as found in post-disaster situations like Hurricane Katrina^27^.

Zika virus (ZIKV), a flavivirus that was relatively unknown until the past decade^28,29^ is transmitted by *Aedes spp* ^30,31^. *Aedes aegypti*, Zika’s main vector^30^, is widely distributed in the tropical lowlands (i.e. coast and Amazon basin) of Ecuador. For instance, a recent study reported the presence of Chikungunya virus (CHIKV) and ZIKV from field collected female *Ae*. *aegypti* in samples from the cities of Esmeraldas (Esmeraldas province), Portoviejo and Manta (Manabi province) in the coast of Ecuador^32^. Other cities where *Ae*. *aegypti* has been reported include Machala (El Oro province, coast)^33–35^ Duran (Guayas province, coast)^36^, Borbón (Esmeraldas province, coast)^37^. Indirect evidence of *Aedes aegypti* distribution is provided by the epidemiological profile of the diseases it transmits. According to the Ministry of Health (MoH) of Ecuador, coastal and Amazon basin provinces reported the highest number of cases for Dengue in year 2016^38^. Finally, a recent study showed that the present-day risk of arbovirus transmission is highest in the coast and Amazon basin^39^.

Zika virus was first isolated from a rhesus macaque in the Zika forest of Uganda in 1947, but it has made headlines since its rapid spread into the Western hemisphere^29^. The spread of the virus has been a cause for concern among international health authorities, as it has been found to be associated with neurological manifestations in both newborns and adults^29^. These neurological complications include microcephaly in infants and Guillain–Barré syndrome (GBS) in adults^29^. Responding to these concerns, on February 1, 2016, the World Health Organization declared the Zika-associated neurological complications to be a Public Health Emergency of International Concern (PHEIC)^40^.

By January 9, 2016, Ecuador reported its first two laboratory-confirmed ZIKV cases^41–43^. Since then, the number of cases has steadily increased across the nation. In this study, we explored the impact that the 2016 earthquake had on the number of cumulative incident autochthonous ZIKV cases in Ecuador.

## Results

### Temporal trend

**Figure 1:**
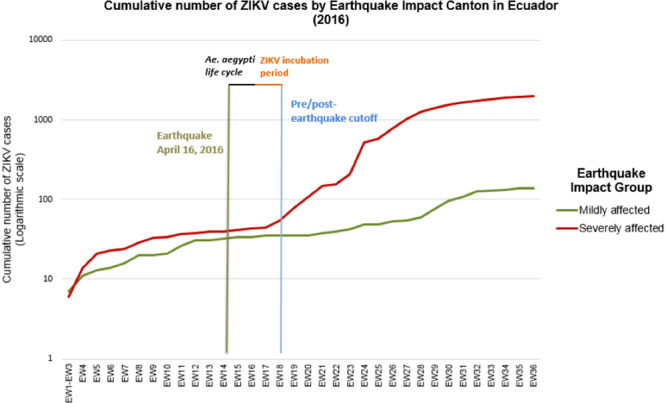
Cumulative number of autochthonous Zika virus (aZIKV) cases by Earthquake Impact Canton in Ecuador. (Cumulative number of aZIKV cases at week 36 for mildly affected cantons = 139; Cumulative number of aZIKV cases at week 36 for severely affected cantons = 1964; cumulative number of aZIKV cases per week detailed in Supplementary Table S1).

As shown in Fig. 1, both severely (n = 26) and mildly (n = 17) affected cantons underwent a steady increase in the number of reported autochthonous ZIKV cases. However, the difference between these two groups of cantons widened significantly during the post-earthquake period (with severely-affected cantons having a significantly higher number of cumulative autochthonous ZIKV cases).

**Figure 2:**
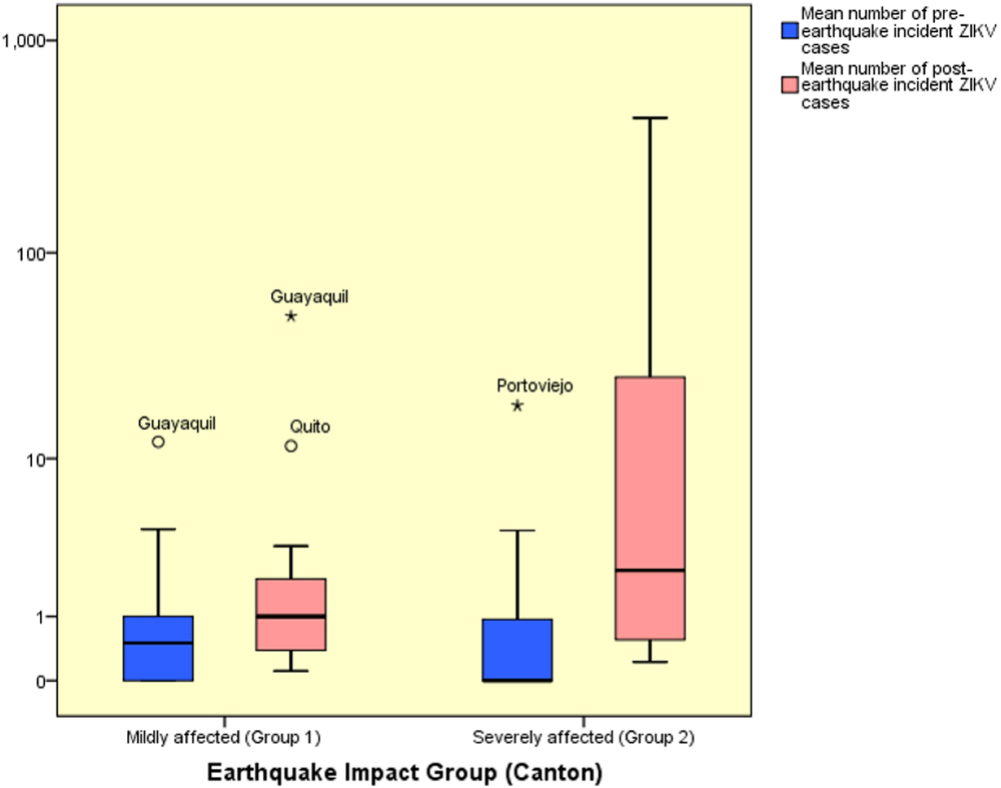
Mean number of incident autochthonous ZIKV cases during the pre- and post-earthquake periods (logarithmic scale).

Before the earthquake (Fig. 2), the average number of weekly reported incident autochthonous ZIKV cases in mildly affected cantons was slightly higher (1.40) as compared to severely affected cantons (1.25) (p = 0.443, Mann-Whitney U test).

However, this relationship was reversed in the post-earthquake period with severely affected cantons having a higher average number of incident autochthonous ZIKV cases (40.19 vs. 4.57) (p = 0.047, Mann-Whitney U Test) (Fig. 2).

### Epidemiological, Socio-economic and climatic characteristics

A comparison of epidemiological, socio-economic and climatic characteristics between mildly affected and severely affected cantons is presented in Table 1. There were no significant differences in pre-earthquake cumulative autochthonous ZIKV incidence rates between severely and mildly affected cantons (mildly affected cantons had slightly higher cumulative autochthonous ZIKV incidence rates). Similar to what was observed for average number of weekly reported incident autochthonous ZIKV cases, this relationship inversed in the post-earthquake period (i.e. cumulative autochthonous ZIKV incidence rates was significantly higher among severely affected cantons as compared to their mildly affected counterparts) (Table 1).

**Table 1:** Epidemiological, Socio-Economic and Climatic characteristics of mildly affected and severely affected cantons.

Characteristic	Mildly affected (Group 1) n = 17	Severely affected (Group 2) n = 26	p-value^+^
**Epidemiological**			
PrEQ cumulative ZIKV incidence rate, *per 100*,*000 pop*	2.01	1.56	0.616^++^
PoEQ cumulative ZIKV incidence rate, *per 100*,*000 pop*	4.42	52.50	0.003^++^*
**Socio-economic**			
School years, mean	9.16	7.88	0.006*
Literacy rate, %	93.3	88.9	0.004*
Poverty rate, %	65.4	81.8	0.002*
Persons per household	3.75	3.99	0.002*
**Climatic**			
PrEQ Max Temp (°C), mean	27.6	29.8	0.262
PoEQ Max Temp (°C), mean	26.3	30.2	0.057**
PrEQ Rainfall (mm), mean	247.4	333.7	0.158
PoEQ Rainfall (mm), mean	61.7	64.9	0.880
PrEQ Rain Days, mean	26.3	28.7	0.050**
PoEQ Rain Days, mean	19.7	19.6	0.985
PrEQ Average Wind (mph), mean	4.19	3.58	0.221
PoEQ Average Wind (mph), mean	5.56	4.98	0.464
PrEQ Pressure (mb), mean	1012.25	1011.56	0.032*
PoEQ Pressure (mb), mean	1014.15	1012.76	<0.001*
PrEQ Humidity (%), mean	82.06	85.45	0.005*
PoEQ Humidity (%), mean	78.77	78.07	0.706
PrEQ Cloud Coverage (%), mean	46.27	67.87	<0.001*
PoEQ Cloud Coverage (%), mean	35.13	57.82	<0.001*
PrEQ UV Index, mean	5.50	5.81	0.499
PoEQ UV Index, mean	5.26	5.92	0.153
PrEQ Sun Hours, mean	96.98	54.15	<0.001*
PoEQ Sun Hours, mean	110.01	72.45	<0.001*
PrEQ Sun Days, mean	2.99	1.38	0.111
PoEQ Sun Days, mean	8.82	8.84	0.996

PrEQ = Pre-earthquake; PoEQ = Post-earthquake; ^++^All p-values correspond to T-test, except where otherwise indicated; ^+^Mann-Whitney test; *p < 0.05; **p = 0.05–0.10.

When comparing severely affected vs. mildly affected cantons, there were significant differences in all socio-economic variables studied including school years, literacy rate, poverty rate and persons per household. However, all these variables were correlated and only persons-per-household was selected to be included in the regression models since it contained the most normally distributed data. Among climatic variables, only pre-earthquake mean pressure, post-earthquake mean pressure, pre-earthquake mean humidity, pre-earthquake mean cloud coverage, post-earthquake mean cloud coverage, pre-earthquake sun hours, and post-earthquake sun hours showed significant differences between the earthquake impact groups (i.e. earthquake severity groups). In addition, post-earthquake mean maximum temperature and pre-earthquake mean rain days had a marginally non-significant association (p = 0.05 – 0.10). After correlation analysis, only (pre- and post-earthquake) maximum temperature, pressure and sun hours were selected to be loaded onto the regression models.

### Multivariate analyses

Multivariate analyses were conducted only on post-earthquake cumulative autochthonous ZIKV incidence rates since there were no significant differences on pre-earthquake cumulative ZIKV autochthonous incidence rates (Table 2). Our data demonstrated that, after controlling for climatic and socio-economic confounders, earthquake impact group (i.e. earthquake severity) was statistically associated with post-earthquake autochthonous ZIKV incidence rates. Areas that were severely affected by the earthquake experienced a significant surge in the incidence of autochthonous Zika infections during the post-earthquake period.

**Table 2:** Stepwise Backward Multiple Negative Binomial Regression for Post-earthquake Cumulative ZIKV Incidence Rates (n = 43).

Variable	Initial Model					Final Model				
	Estimate	SE	95% CI		p-value	Estimate	SE	95% CI		p-value
			LB	UB				LB	UB	
Earthquake impact group	2.587	0.827	0.964	4.209	0.002*	1.432	0.593	0.269	2.595	0.016
Persons per household	−1.076	1.368	−3.757	1. 605	0.431	—	—	—	—	—
PrEQ Max Temp	−0.269	0.234	−0.727	0.189	0.250	−0.150	0.058	−0.263	−0.037	0.009
PoEQ Max Temp	0.174	0.254	−0.324	0.671	0.495	—	—	—	—	—
PrEQ Pressure	−0.253	0.885	−1.988	1. 481	0.775	—	—	—	—	—
PoEQ Pressure	−0.794	0.747	−2.258	0.670	0.288	−1.344	0.273	−1.878	−0.809	<0.001
PrEQ Sun Hours	0.028	0.036	−0.043	0.098	0.446	—	—	—	—	—
PoEQ Sun Hours	−0.009	0.035	−0.078	0.059	0.787	—	—	—	—	—
Dispersion	1.551	0.357	0.988	2.437	—	1.667	0.382	1.064	2.612	—

PrEQ = Pre-earthquake; PoEQ = Post-earthquake; *p < 0.05.

### Spatial analysis

**Figure 3:**
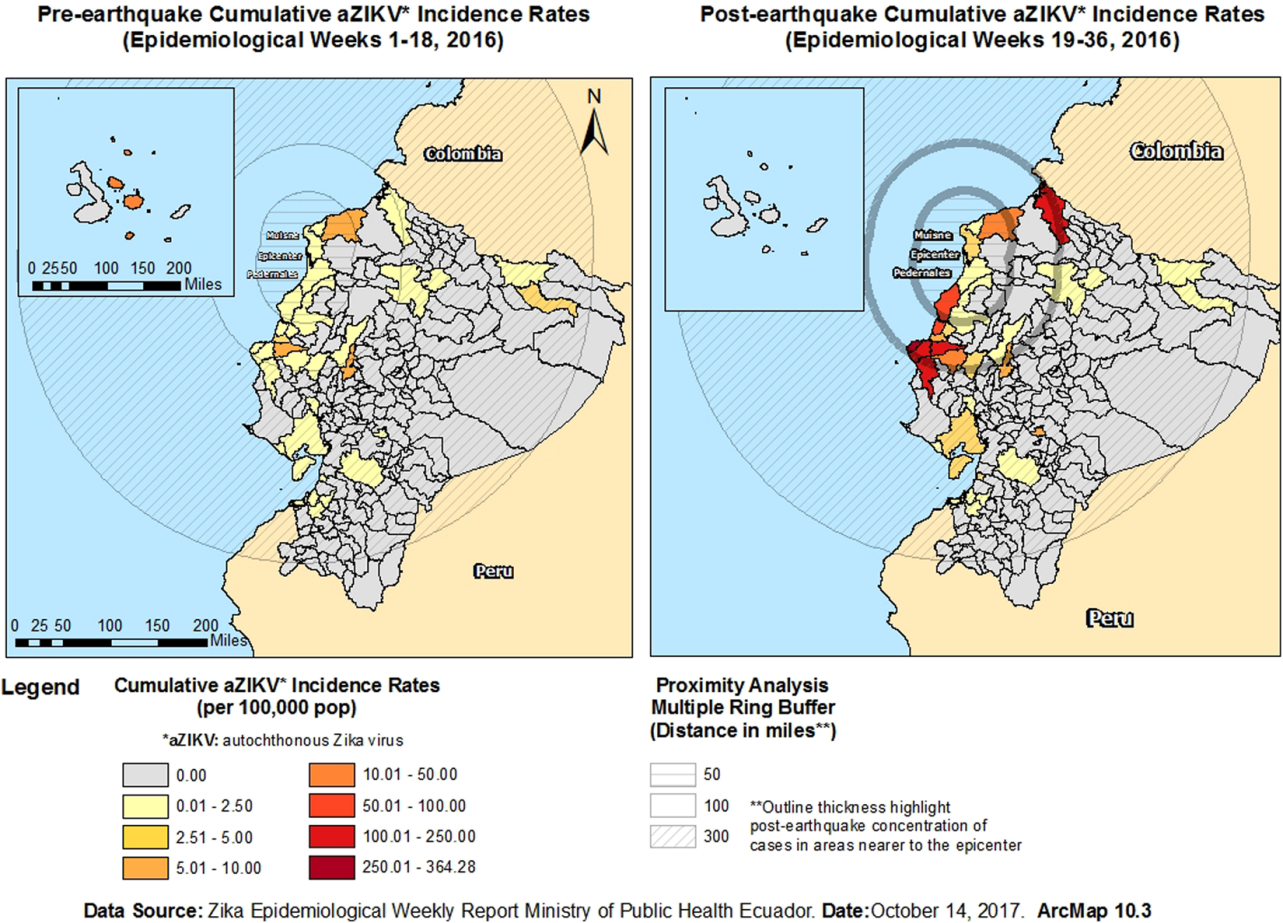
Epicentre Multiple Ring Buffer Analysis by Pre- and Post-earthquake cumulative ZIKV incidence rates. Maps created using ArcMap 10.3 (http://desktop.arcgis.com/en/arcmap/).

A Multiple Ring Buffer Analysis was conducted to visualize pre- and post-earthquake cumulative ZIKV incidence rates in severely and mildly affected cantons and their distance/relationship to 50-, 100- and 300-mile buffers created around the earthquake epicentre and Muisne/Pedernales (Fig. 3). Before the earthquake, cantons with the highest cumulative autochthonous ZIKV incidence rates were located both at close and mid-to-far distance from the epicentre and/or Muisne/Pedernales; however, after the earthquake, the cantons with the highest cumulative autochthonous ZIKV incidence rates were relatively closer to the epicentre and/or Muisne/Pedernales (Fig. 3).

## Discussion

Our study found a significant increase in the cumulative number of autochthonous ZIKV cases between study periods (i.e. cases raised from 89 in the pre-earthquake period to 2,103 during the post-earthquake period; see Supplementary Table S1). This was especially evident in the cantons that were severely affected by the earthquake (n = 25) as compared to those mildly affected (n = 17). Prior to the earthquake, the difference in the cumulative autochthonous ZIKV incidence rate between severely and mildly affected cantons was not significant and slightly higher in the latter group (i.e. 1.56 vs. 2.01 per 100,000 pop). After the earthquake, the severely affected cantons had a significant higher cumulative autochthonous ZIKV incidence rate as compared to those mildly affected (i.e. 52.50 vs. 4.42 per 100,000 pop, respectively). Our preliminary research had already suggested an increase in post-earthquake incident ZIKV cases in Ecuador during the period January-July 2016^44^. A later study confirmed this observation^45^. However, neither of these studies controlled for socioeconomic and climatic variables nor did they evaluate the impact of climatic context on this phenomenon, which might confound the observed associations. In the present study, we addressed previous limitations by evaluating the impact of earthquake intensity on cumulative ZIKV incidence rates during equally long, mosquito life and incubation period-adjusted pre- and post-earthquake periods among mildly and severely affected cantons within their respective socioeconomic and climatic characteristics. To the best of our knowledge, this is the first study to address post-disaster vector-borne disease transmission dynamics using this methodology.

**Figure 4:**
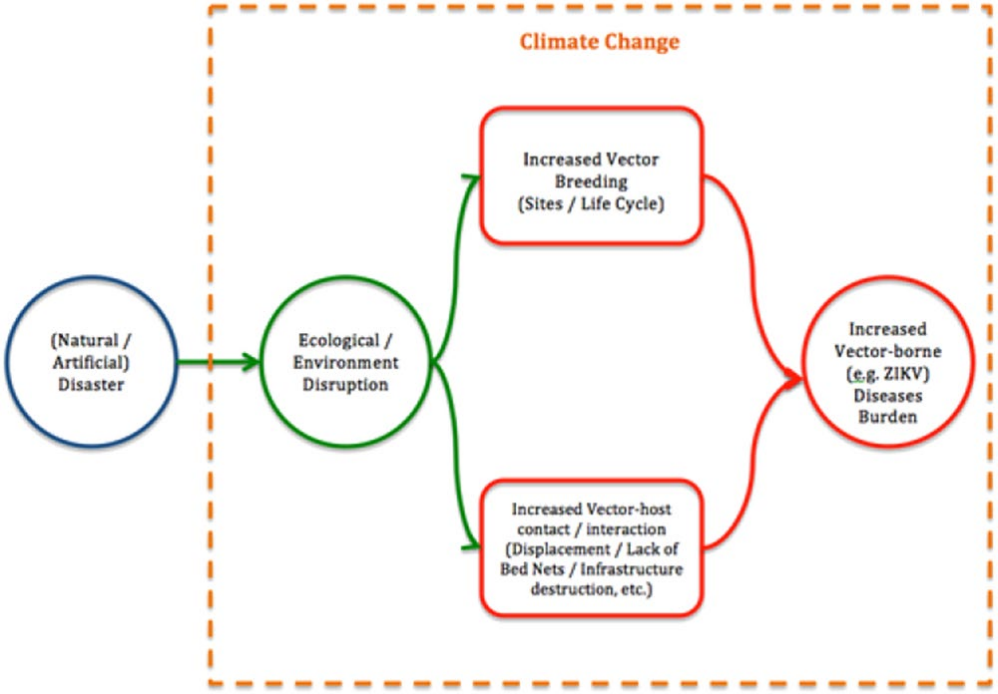
Conceptual model of the relationship between disaster-induced ecological or environmental disruption and increased vector-borne disease burden.

This increase in ZIKV cases could be due to a multifactorial event. The earthquake in Ecuador resulted in a lot of destruction and building debris^10,11^. These partial structures could have stored rainwater that could act as a mosquito breeding ground^20^. Due to the building destruction, many people lost homes and had to resort to living in camps. There were a maximum of 29 official camps (recognized as such by the Coordinating Ministry of Security of Ecuador)^46^ at any given point after the earthquake in addition to several “unofficial” camps. Although vector-borne strategies were implemented in the official camps, the use of bed-nets (which might be useful in the control of malaria) had limited impact in Zika virus transmission given the biting behaviour of aedine mosquitoes. In addition, it is possible that the unofficial camps did not have access to the same levels of vector-control strategies further increasing the risk of Zika transmission in affected populations. Moreover, it is likely that people, especially those living in displaced population camps, spend more time outdoors, which may increase chance of exposure to mosquito bites, especially if mosquitoes are breeding in the camp areas. A recent study showed that time spent outdoors, and the proportion of people spending more time outdoors in a susceptible population, may affect Zika transmission and therefore Zika epidemic size^47^. Similarly, these camps may have lacked the typical public utilities and sanitation that could have led to conditions like those resulting in the increase in malaria post-earthquake in Haiti^17^. Altogether, these conditions may lead to an increased likelihood of mosquito-susceptible host interaction (Fig. 4).

*A*. *aegypti* tends to feed during the daytime and prefer clean water^48^. Since people are more active and ambulant during the daytime, they are more exposed to mosquitoes. Also, during an earthquake, water and sanitation infrastructures are severely affected and people are forced to store water for its consumption. The water collected by people in containers could have further attracted the mosquitoes for their breeding purposes. The earthquake could have disrupted the routine insecticide prevention control schedule resulting in an unopposed upsurge in mosquito population.

Climatic variables are known to affect vector-borne diseases transmission. The rise in vector-borne diseases such as dengue and malaria has been found to have a direct relationship with the occurrence of strong El Niño events^49–51^. Similarly, vector-borne diseases have been found to be associated with Surface Sea Temperatures and the Tropical South Atlantic^52^. Furthermore, climate change and changing temperatures, humidity and/or rainfall have been associated with vector-borne disease transmission^2–7,53^. For example, climate change has been estimated to slow the reduction of vector-borne diseases in China^54^. Therefore, this post-earthquake scenario, in the context of permissive environmental conditions, may have led to an ecological disruption leading to increased ZIKV transmission upsurge. Despite strong association of vector-borne diseases and natural disasters, increased post-earthquake incidence has not been reported for all flaviviruses or in all post-disaster scenarios. For instance, the incidences of Japanese encephalitis, along with other vector-borne diseases like visceral leishmaniasis and malaria, did not increase after the Wenchuan earthquake in China in 2008^55^.

Such increased public health burden from vector-borne diseases is more likely to occur if the disaster occurs in the appropriate climatic context. In this study we observed that post-earthquake ZIKV cases increased not only in severely affected cantons but also in those areas where the pre-earthquake temperature and the post-earthquake pressure were lower. This is extremely important as temperature and rainfall, which in turn is affected by pressure, are factors known to be associated with the breeding of mosquito and other vectors^2–4,8^. Thus, earthquakes and other disasters occurring in the appropriate climatic context could lead to outbreaks of vector-borne diseases such as ZIKV and other viral, bacterial and parasitic diseases. We propose a conceptual model of the relationship between different factors/determinants responsible for the observed increased vector-borne disease burden in post-disaster scenarios (Fig. 4).

To prevent future increases of ZIKV cases, especially after the occurrence of a natural disaster, there is the need to focus on a few key features: prevention, detection, and control. We need to ensure that regularly scheduled insecticide use for maintaining mosquito populations does not get interrupted. In addition, there may be a need to increase the spraying of insecticides in the aftermath of a natural (or artificial) disaster. As ZIKV can be transmitted through sexual contact or blood transfusion in addition to the mosquito vector, population awareness campaigns are needed regarding the different risk factors and transmission prevention strategies^56^. Surveillance of ZIKV also needs to be maintained in order to detect a rise in transmission rates of ZIKV cases in order to allow for timely interventions.

This study highlights the importance of appropriate vector-borne disease control following an earthquake, and possibly other post-disaster scenarios like tornado- or hurricane-affected areas, especially in the appropriate climatic context, including changing temperatures. In this natural experiment, conditions in Ecuador created the “perfect storm” for an upsurge of Zika virus cases but it may be possible that, due to changing global temperatures, similar conditions may accrue in northern and further southern latitudes where these diseases currently do not pose a significant threat but which may do so if the “perfect storm” occurs. The Intergovernmental Panel on Climate Change (IPCC) deems very likely that an overall global decrease in the number of cold days and nights with an overall increase in the number of warm days and nights has occurred with such changes also likely occurring on a continental scale in North America, Europe and Australia^1^. Moreover, recent studies suggest that displacement of climatic isoclines is faster at higher latitudes, which may lead to new ecological niches and therefore novel species assemblages^57^. Therefore, this study provides critical information that may help prevent significant public health burden in largely populated areas of North America, Europe and Australia where increasing temperatures may fall within the range for conducive mosquito (or other vector) breeding.

The strengths of this study are multiple and include the fact that the data was nationally collected by Ecuador’s Ministry of Public Health’s Epidemiological Surveillance System, which relied on accurate diagnostic methods (RT-PCR) for Zika infection. In addition, our results were adjusted by socio-economic and climatic variables and used a complex approach to evaluate the impact of earthquake intensity on incident ZIKV rates. The equation for the study of vector-borne disease transmission dynamics in post-disaster scenarios is another important strength and contribution of this study. Our study was limited by the amount of available data. Only canton-level aggregated weekly totals of ZIKV cases (and not individual data on the people affected) were available. Additional person-level data might have provided further insight on whether there were any other predisposing factors that could have contributed to a rise in ZIKV cases in addition to the earthquake. Natural disasters are unpredictable and potentially disastrous. With the understanding that post-disaster outbreaks of ZIKV, or other vector-borne diseases, are possible, especially in the context of warm(ing) temperatures which are likely to occur in largely populated regions of North America and Europe, it is important to become more vigilant in the prevention of future disaster-induced outbreaks.

## Methods

### Study population

**Figure 5:**
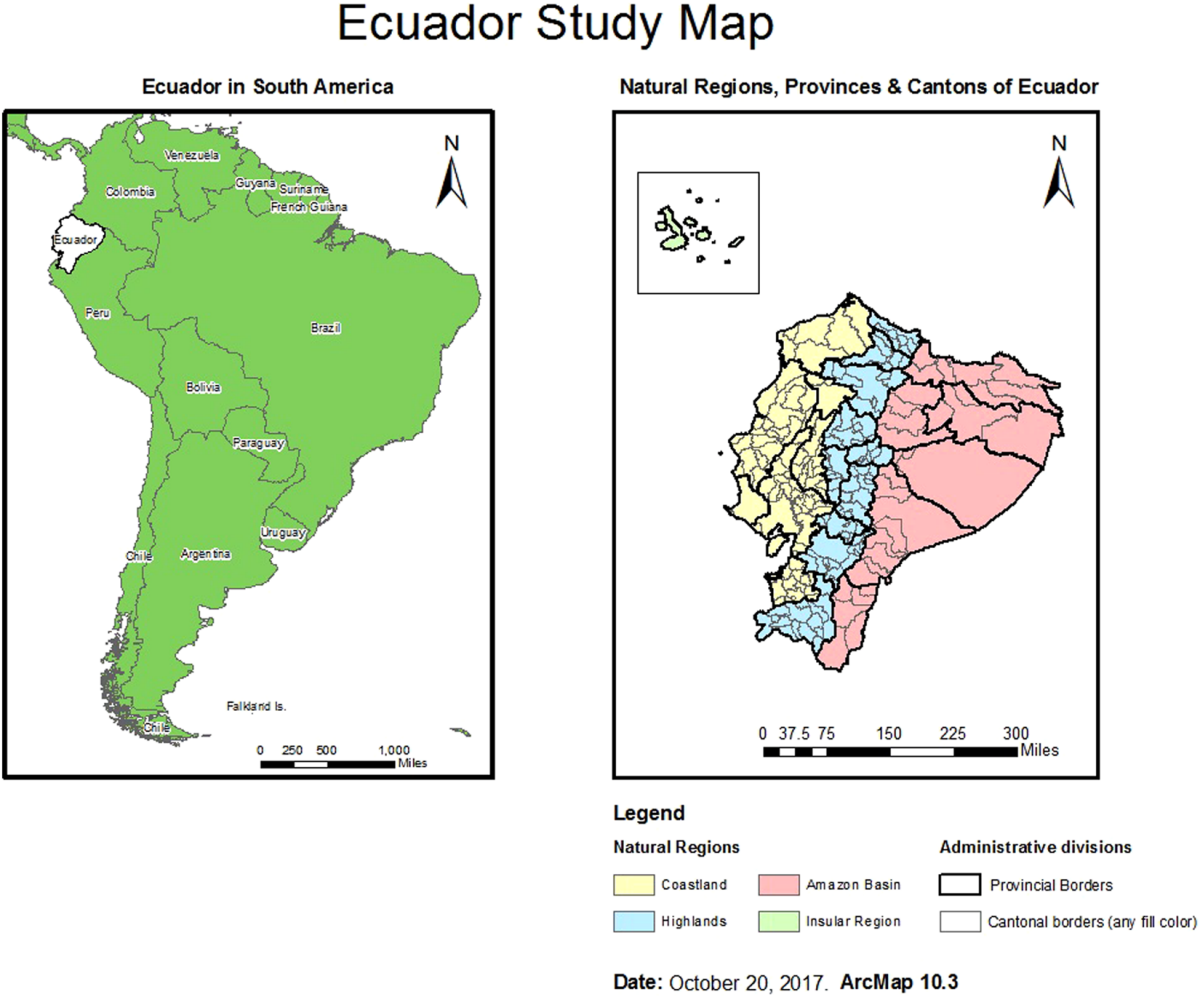
Ecuador Study Map. Maps created using ArcMap 10.3 (http://desktop.arcgis.com/en/arcmap/).

Ecuador is a democratic republic located in the north-western region of South America (Fig. 5). Ecuador is administratively divided into Regions, Provinces and Cantons, which would be equivalents to Regions, Counties and Districts in England, or to States, Counties and Townships in the United States. For this study, all incident autochthonous ZIKV cases reported by the MoH of Ecuador between Epidemiological Week 1 and 36 (2016) were included in the analysis. Zika cases were diagnosed using RT-PCR techniques and were reported aggregated at the canton level, as per standard MoH procedures. A total of 2,103 autochthonous ZIKV cases were reported during the study period in 43 cantons, out of 229 cantons, of Ecuador. Cantons not reporting ZIKV cases were excluded from the study since it would not be possible to evaluate the effect of earthquake on Zika transmission in such cantons. The number of cumulative autochthonous ZIKV cases for the study period ranged from one in 16 cantons (Cuenca, Chunchi, Huaquillas, Pasaje, Santa Rosa, Gral. Antonio Elizalde, Playas, La Joya de los Sachas, Cayambe, Mejia, Lago Agrio, Atacames, Muisne, Rioverde, Pajan, Tosagua) to 827 in Manta canton.

### Study Design

A natural experiment-based, ecological study was conducted to compare the number of reported ZIKV cases before and after a M7.8 earthquake registered on April 16, 2016 in Ecuador. Nationwide, ZIKV data (aggregated at the canton level, an administrative division of the country) were collected from the Weekly Epidemiological Zika Virus Reports published by the Ministry of Health of Ecuador. The total number of cumulative incident ZIKV cases reported by canton and per epidemiological week (EW) was compiled and analysed. May 8, 2016 was used to differentiate pre and post-earthquake cases based on the criteria outlined in Equation (1) (note: this equation could potentially be applied to other scenarios and pathogens).

1 $$(Zika\,Earthquake)\,Disaster\,Cutoff\,Date=\{DD+VLC+PIP\}WE(Z)R$$

Where DD stands for Disaster (Earthquake) Date, VLC stands for Vector’s Life Cycle in days, PIP stands for Pathogen Incubation Period in days, WEZR stands for Weekly Epidemiological (Zika) Report, and where the square bracket signifies that the Zika Earthquake Cutoff Date will be approximated to the nearest WEZR date. Then, April 16, 2016 (Earthquake date) + 10 days (*Aedes aegypti* incubation period) = April 26, 2016 + 12 days (maximum reported ZIKV incubation period) = May 8th. Therefore, all cases occurring up to EW 18 (i.e. up to May 7^th^) were considered pre-earthquake infections whereas all cases occurring from EW19 (i.e. from May 8^th^ onwards) were designated as post-earthquake infections. In order to control for temporal variations, the study period spans 18 pre-earthquake weeks (i.e. until EW 18 in 2016) and 18 post-earthquake weeks (i.e. from EW 19 until EW 36 in 2016). Climatic data including average rainfall, rain days, temperature, pressure, average wind speed, humidity, cloud coverage, UV index, sun hours, and sun days were collected from an online database (data provided by https://www.worldweatheronline.com). Climatic variables were aggregated as pre- and post-earthquake averages to account for their effect on incident cases (i.e. to assess the effect of average climatic conditions during the pre- and post-earthquake periods on cumulative autochthonous incident ZIKV cases). Canton-level socio-economic data including schooling years, literacy rate, poverty rate and number of persons per household were gathered from Ecuador’s VII Population Census (available at http://www.inec.gob.ec).

Cantons in Ecuador were classified according to earthquake impact (i.e. earthquake impact group) into severely affected or mildly affected as per the USGS Modified Mercalli Intensity (MMI) scale-based Community Internet Intensity Map (available at https://earthquake.usgs.gov/earthquakes/eventpage/us20005j32#map). Briefly, MMI scale ranges from I – not felt – to X – extreme. In the aftermath of the April 2016 earthquake in Ecuador, reported intensity ranged from I – not felt – to VIII – severe. Cantons in Ecuador were classified into two groups: 1) mildly affected (n = 17), those with reported intensity between I (not felt) and V (moderately felt); and, 2) severely affected (n = 26), those with reported intensity between VI (strong) and VIII (severe).

### Statistical Analysis

Data was analysed using SPSS v22 (IBM SPSS Statistics v22). All statistical analyses were two-tailed. The population of each canton was gathered from Ecuador’s VII Population Census. Cumulative autochthonous ZIKV incidence rates (per 100,000 pop) were calculated. Cumulative autochthonous ZIKV incidence rates at epidemiological week 18 and cumulative ZIKV incidence rates for epidemiological weeks 19–36 were calculated. Pre- and post-earthquake cumulative autochthonous ZIKV incidence rate comparisons by earthquake impact group were performed using the Mann-Whitney test. Similarly, bivariate analyses to compare socio-economic (4 variables) and climatic variables (20 variables) between earthquake impact groups were conducted to identify other significantly associated variables. Pearson’s correlations were utilized to identify correlated variables (17 redundant variables were excluded from further analyses; that is, 7 of these variables were included in multivariate analyses). Both pre- and post-earthquake measurements were used for selected variables even if only one of those measurements was chosen following bivariate and correlation analyses. Based on these considerations, the regression models included only the following variables: persons per household, pre-earthquake mean maximum temperature, post-earthquake mean maximum temperature, pre-earthquake mean pressure, post-earthquake mean pressure, pre-earthquake mean sun hours, and post-earthquake mean sun hours. In order to control for data over-dispersion, a negative binomial model was fit using SAS 9.4 software to evaluate the association between earthquake impact group and post-earthquake cumulative ZIKV incidence rate.

Spatial models were also constructed to visualize the observed differences. Earthquake epicentre coordinates were obtained from the United States Geological Service (available at https://earthquake.usgs.gov/earthquakes/eventpage/us20005j32#origin?source = at&code=at00o5r3xd). In addition, geographic coordinates for the two main population settlements affected by the earthquake (i.e. Muisne in Esmeraldas province and Pedernales in Manabí province) were also obtained. Based on these coordinates, a KMZ file was generated using Google Earth Pro 7.1.8.3036. The KMZ file was imported into ArcMap 10.3 for further spatial analysis. Ecuador shapefiles were downloaded from an online server (DIVA GIS, available at http://www.diva-gis.org/). A vector-based model was created to represent pre- and post-earthquake cumulative ZIKV incidence rates per canton. Finally, a multiple ring buffer analysis was conducted to visually demonstrate the spatial correlation between pre- and post-earthquake cumulative ZIKV incidence rates and areas located within 50, 100, and 300 miles from the earthquake epicentre or from the main two population settlements affected by the earthquake (i.e. Muisne in Esmeraldas province and Pedernales in Manabí province). Ring buffer sizes were determined considering that tertiles were needed to distinguish different distances and that the distance between the Epicenter area to the farthest large city to have felt the earthquake with an intensity of at least IV (Loja, Loja Province) is 300 miles.

### Data availability

The data that support the findings of this study are available on request from the corresponding author [R.I.]. The data are not publicly available due to third-party restrictions.

## Electronic supplementary material

Supplementary Table S1

## References

[1] IPCC in Managing the Risks of Extreme Events and Disasters to Advance Climate Change Adaption (eds Field, C. B. *et al*.) 109–230 (Cambridge Univ. Press, 2012). 10.1136/jech-2012-201045 22766781

[2] Jia P (2017). How does the dengue vector mosquito *Aedes albopictus* respond to global warming?. Parasit. Vectors.

[3] Wang X, Zhao XQ (2017). A Malaria Transmission Model with Temperature-Dependent Incubation Period. Bull. Math. Biol..

[4] Malik A (2017). Assessing spatio-temporal trend of vector breeding and dengue fever incidence in association with meteorological conditions. Environ. Monit. Assess..

[5] Contestabile M (2014). Health: Dengue Drivers. Nat. Clim. Change.

[6] Semenza JC, Domanović D (2013). Blood supply under threat. Nat. Clim. Change.

[7] Caminade C, Jones AE (2016). Epidemiology: Malaria in a warmer West Africa. Nat. Clim. Change.

[8] Kumari R, Joshi PL, Lal S, Shah W (2009). Management of malaria threat following tsunami in Andaman & Nicobar Islands, India and impact of altered environment created by tsunami on malaria situation of the islands. Acta Trop..

[9] Lingling Y (2016). The 16 April 2016, MW 7.8 (MS 7.5) Ecuador earthquake: A quasi-repeat of the 1942 MS 7.5 earthquake and partial re-rupture of the 1906 MS 8.6 Colombia–Ecuador earthquake. Earth Planet. Sci. Lett..

[10] Cordero-Reyes AM (2016). Natural disaster management: experience of an academic institution after a 7.8 magnitude earthquake in Ecuador. Public Health.

[11] International Organization for Migration (IOM). Displaced Ecuador Earthquake Survivors Face Lack of Water, Sanitation, Funding [online]. Available at https://www.iom.int/news/displaced-ecuador-earthquake-survivors-face-lack-water-sanitation-funding (accessed February 22, 2017) (IOM, 2016).

[12] Jafari N, Shahsanai A, Memarzadeh M, Loghmani A (2011). Prevention of communicable diseases after disaster: A review. J. Res. Med. Sci..

[13] Kinston W, Rosser R (1974). Disaster: effects on mental and physical state. J. Psychosom. Res..

[14] Bland SH, O’Leary ES, Farinaro E, Jossa F, Trevisan M (1996). Long-term psychological effects of natural disasters. Psychosom. Med..

[15] Chin CS (2011). The origin of the Haitian cholera outbreak strain. N. Engl. J. Med..

[16] Eisenberg MC, Kujbida G, Tuite AR, Fisman DN, Tien JH (2013). Examining rainfall and cholera dynamics in Haiti using statistical and dynamic modeling approaches. Epidemics.

[17] Gharbi M (2012). Chloroquine-resistant malaria in travelers returning from Haiti after 2010 earthquake. Emerg. Infect. Dis..

[18] Polonsky J (2013). Public Health Surveillance After the 2010 Haiti Earthquake: the Experience of Médecins Sans Frontières. PLoS. Curr..

[19] Mason J, Cavalie P (1965). Malaria Epidemic in Haiti Following a Hurricane. Am. J. Trop. Med. Hyg..

[20] Saleeza SN, Norma-Rashid Y, Azirun MS (2013). Mosquito species and outdoor breeding places in residential areas in Malaysia. Southeast Asian J. Trop. Med. Public Health.

[21] Moise IK, Brown KS, Riegel C, Kalipeni E, Ruiz MO (2012). Geographic Assessment of Unattended Swimming Pools in Post-Katrina New Orleans, 2006–2008. Annals of the Association of American Geographers.

[22] Caillouet KA, Carlson JC, Wesson D, Jordan F (2008). Colonization of abandoned swimming pools by larval mosquitoes and their predators following Hurricane Katrina. J. Vector Ecol..

[23] Reisen WK, Takahashi RM, Carroll BD, Quiring R (2008). Delinquent mortgages, neglected swimming pools, and West Nile virus, California. Emerg. Infect. Dis..

[24] Samson DM (2015). New baseline environmental assessment of mosquito ecology in northern Haiti during increased urbanization. J. Vector Ecol..

[25] Marquetti Fernandez MC, Fuster Callaba CA, Estevez Torres G, Somarriba Lopez L (2011). [Contributions made by the Cuban advisory work to the entomological surveillance in Haiti]. Rev. Cubana Med. Trop..

[26] Saenz R, Bissell RA, Paniagua F (1995). Post-disaster malaria in Costa Rica. Prehosp. Disaster Med..

[27] Moise IK, Ruiz MO (2016). Hospitalizations for Substance Abuse Disorders Before and After Hurricane Katrina: Spatial Clustering and Area-Level Predictors, New Orleans, 2004 and 2008. Prev Chronic Dis.

[28] Hennessey M, Fischer M, Staples JE (2016). Zika Virus Spreads to New Areas - Region of the Americas, May 2015-January 2016. MMWR Morb. Mortal. Wkly. Rep..

[29] Sikka V (2016). The Emergence of Zika Virus as a Global Health Security Threat: A Review and a Consensus Statement of the INDUSEM Joint working Group (JWG). J. Glob. Infect. Dis..

[30] Saldaña MA (2017). Zika virus alters the microRNA expression profile and elicits an RNAi response in Aedes aegypti mosquitoes. PLoS Negl. Trop. Dis..

[31] Azar SR (2017). Differential Vector Competency of Aedes albopictus Populations from the Americas for Zika Virus. Am. J. Trop. Med. Hyg..

[32] Cevallos V (2017). Zika and Chikungunya virus detection in naturally infected Aedes aegypti in Ecuador. Acta Trop..

[33] Heydari N (2017). Household Dengue Prevention Interventions, Expenditures, and Barriers to Aedes aegypti Control in Machala, Ecuador. Int. J. Environ. Res. Public Health.

[34] Quintero J (2014). Ecological, biological and social dimensions of dengue vector breeding in five urban settings of Latin America: a multi-country study. BMC Infect. Dis..

[35] Stewart Ibarra AM (2013). Dengue vector dynamics (Aedes aegypti) influenced by climate and social factors in Ecuador: implications for targeted control. PLoS One.

[36] Qualls WA (2016). Movement of Aedes aegypti following a sugar meal and its implication in the development of control strategies in Durán, Ecuador. J. Vector Ecol..

[37] Schafrick NH, Milbrath MO, Berrocal VJ, Wilson ML, Eisenberg JN (2013). Spatial clustering of Aedes aegypti related to breeding container characteristics in Coastal Ecuador: implications for dengue control. Am. J. Trop. Med. Hyg..

[38] Granda, J. Dirección Nacional de Vigilancia Epidemiológica, Ministerio de Salud Pública del Ecuador. *Anuario de Vigilancia Epidemiológica 1994–2016 Enfermedades Transmitidas por Vectores* https://public.tableau.com/profile/vvicentee80#!/vizhome/EnfermeddaesTropicales_vectoriales-2014/ANUARIO (Accessed October 7, 2017) (2017).

[39] Escobar LE (2016). Declining Prevalence of Disease Vectors Under Climate Change. Sci. Rep..

[40] World Health Organization. *WHO Director-General summarizes the outcome of the Emergency Committee regarding clusters of microcephaly and Guillain-Barré syndrome* http://www.who.int/mediacentre/news/statements/2016/emergency-committee-zika-microcephaly/en/ (accessed March 7, 2017) (2016).

[41] World Health Organization. *Zika virus infection – Guyana*, *Barbados and Ecuador* http://www.who.int/csr/don/20-january-2016-zika-guyana-barbados-ecuador/en/ (accessed February 22, 2017) (2016).

[42] Pan American Health Organization and World Health Organization. *Zika-Epidemiological Report- Ecuador* http://www.paho.org/hq/index.php?option=com_docman&task=doc_view&gid=35027&Itemid=270 (accessed February 22, 2017) (2016).

[43] Ministerio de Salud Pública del Ecuador. *Ecuador confirma dos casos importados de Zika* http://www.salud.gob.ec/ecuador-confirma-dos-casos-importados-de-zika/ (accessed March 3, 2017) (2016).

[44] Reina Ortiz, M. *et al*. Post-earthquake Zika Virus surge in tropical lowlands of Ecuador: an analysis of population-based incident cases from January to July 2016. Session 27, LB-5150 (American Society of Tropical Medicine and Hygiene Annual Meeting, Atlanta, GA, 2016).

[45] Vasquez D (2017). Impact of the 2016 Ecuador Earthquake on Zika Virus Cases. Am. J. Public Health.

[46] Ministerio Coordinador de Seguridad del Ecuador. *Albergues Oficiales Implementados Sismo 7.8 del 16 de abril del 2016* http://cifras.seguridad.gob.ec/albergues/index.php (accessed June 17, 2017) (2017).

[47] Ajelli M (2017). Host outdoor exposure variability affects the transmission and spread of Zika virus: Insights for epidemic control. PLoS Neglected Tropical Diseases.

[48] Morrison, A. C., Zielinski-Gutierrez, E., Scott, T. W. & Rosenberg, R. Defining Challenges and Proposing Solutions for Control of the Virus Vector Aedes aegypti (2008). *PLoS Med*. **5**, e68, 10.1371/journal.pmed.0050068 (2008). 10.1371/journal.pmed.0050068 PMC2267811 18351798

[49] Poveda G (2001). Coupling between annual and ENSO timescales in the malaria-climate association in Colombia. Environ. Health. Perspect..

[50] Kovats RS, Bouma MJ, Hajat S, Worrall E, Haines A (2003). El Nino and health. Lancet.

[51] Fuller DO, Troyo A, Beier JC (2009). El Nino Southern Oscillation and vegetation dynamics as predictors of dengue fever cases in Costa Rica. Environ. Res. Lett..

[52] Cash BA (2013). Malaria epidemics and the influence of the tropical South Atlantic on the Indian monsoon. Nat. Clim. Change.

[53] Costello A (2011). March of the maladies. Nat. Clim. Change.

[54] Hodges M (2014). Delays in reducing waterborne and water-related infectious diseases in China under climate change. Nat. Clim. Change.

[55] Zhang S (2013). Incidence of Japanese encephalitis, visceral leishmaniasis and malaria before and after the Wenchuan earthquake, in China. Acta Trop..

[56] Broutet N (2016). Zika Virus as a Cause of Neurologic Disorders. N. Engl. J. Med..

[57] Ordonez A, Williams JW, Svenning JC (2016). Mapping climatic mechanisms likely to favour the emergence of novel communities. Nat. Clim. Change.

