# Supplementary material for: Post-earthquake Zika virus surge: Disaster and public health threat amid climatic conduciveness
Source: Sci Rep. 2017 Nov 13;7:15408. doi: 10.1038/s41598-017-15706-w (PMC5684400; doi:10.1038/s41598-017-15706-w)
Supplement: Supplementary file 1 — Supplementary Table S1 [file 41598_2017_15706_MOESM1_ESM.pdf]

Post-earthquake Zika virus surge: Disaster and public health threat amid climatic conduciveness

Miguel Reina Ortiz[1,2], Nicole K. Le[3], Vinita Sharma[4], Ismael Hoare[1], Edy Quizhpe[5,6], Enrique Teran[6], Eknath Naik[7], Hamisu M. Salihu[8] & Ricardo Izurieta[1,*]

[1] Department of Global Health, College of Public Health, University of South Florida, Tampa, FL, USA.
[2] Fundación Raíces. Esmeraldas, Ecuador.
[3] Morsani College of Medicine, University of South Florida, Tampa, FL, USA.
[4] Department of Community and Family Health, College of Public Health, University of South Florida, Tampa, FL, USA.
[5] Ministerio de Salud Pública del Ecuador, Quito, Ecuador.
[6] Colegio de Ciencias de la Salud, Universidad San Francisco de Quito, Quito, Ecuador
[7] Emergency Department, James A Haley VA Hospital, Tampa, FL, USA.
[8] Department of Family and Community Medicine, Baylor College of Medicine, Houston, TX.

* Correspondence to rizuriet@health.usf.edu

Supplementary materials

Supplementary Table S1

Cumulative autochthonous Zika cases by Earthquake Impact Group and by Epidemiological Week.

	Earthquake Impact Group	
	Mildly affected	Severely affected
caZIKV E. Week 3	7	6
caZIKV E. Week 4	11	14
caZIKV E. Week 5	13	21
caZIKV E. Week 6	14	23
caZIKV E. Week 7	16	24
caZIKV E. Week 8	20	29
caZIKV E. Week 9	20	33
caZIKV E. Week 10	21	34
caZIKV E. Week 11	26	37
caZIKV E. Week 12	31	38
caZIKV E. Week 13	31	40
caZIKV E. Week 14	32	40
caZIKV E. Week 15	34	41
caZIKV E. Week 16	34	43
caZIKV E. Week 17	35	44
caZIKV E. Week 18	35	54
caZIKV E. Week 19	35	79
caZIKV E. Week 20	35	108
caZIKV E. Week 21	38	147
caZIKV E. Week 22	40	156
caZIKV E. Week 23	42	210
caZIKV E. Week 24	49	515
caZIKV E. Week 25	49	586
caZIKV E. Week 26	53	780
caZIKV E. Week 27	55	1026
caZIKV E. Week 28	59	1256
caZIKV E. Week 29	76	1405
caZIKV E. Week 30	96	1540
caZIKV E. Week 31	107	1669

caZIKV E. Week 32	127	1730
caZIKV E. Week 33	129	1817
caZIKV E. Week 34	131	1888
caZIKV E. Week 35	138	1931
caZIKV E. Week 36	139	1964

caZIKV = Cumulative Autochthonous Zika virus cases
